# Application of Lactoferrin and *α*1-Antitrypsin in Gingival Retention Fluid to Diagnosis of Periodontal Disease

**DOI:** 10.1155/2018/4308291

**Published:** 2018-11-07

**Authors:** Ryosuke Koshi, Kazuhiko Kotani, Mariko Ohtsu, Naoto Yoshinuma, Naoyuki Sugano

**Affiliations:** ^1^Department of Periodontology, Nihon University School of Dentistry, Tokyo, Japan; ^2^Division of Community and Family Medicine, Jichi Medical University, Tochigi, Japan; ^3^Department of Pathology, Nihon University School of Dentistry, Tokyo, Japan

## Abstract

**Objectives:**

Periodontal disease is prevalent and has an inflammation associated with not only oral but also systemic pathologies. The diagnosis by biomarkers is required for clinical practice on periodontal disease. The lactoferrin and *α*1-antitrypsin were both inflammation-related molecules. The present study investigated the relationship between the periodontal status and the two biomarkers in gingival retention fluid (GRF).

**Patients and Methods:**

In 63 subjects with periodontitis, the GRF was sampled from maxillary anterior gingiva using a microbrush for 30 seconds. The lactoferrin and *α*1-antitrypsin levels in GRF were measured by an enzyme-link solvent immunoassay. Periodontal status was evaluated by probing pocket depth (PD) and bleeding on probing (BOP).

**Results:**

There was a higher level of these biomarkers in saliva (median (ng/mL), lactoferrin: 3611.9, *α*1-antitrypsin: 4573.3) than in GRF (lactoferrin: 61.0, *α*1-antitrypsin: 54.7). There was a mild-to-moderate but significantly positive correlation in lactoferrin or *α*1-antitrypsin between GRF and saliva. There was a positively mild-to-moderate accuracy (area under the curve: 0.60–0.81) of lactoferrin or *α*1-antitrypsin in GRF or in saliva to distinguish the severity of periodontal status. The cutoff level (ng/mL) of lactoferrin in GRF for detecting ≥30% of PD ≥ 4 mm (moderate periodontitis) was 68.6 and for detecting ≥20% of BOP (clinically active periodontitis) was 61.2. The cutoff level (ng/mL) of *α*1-antitrypsin in GRF for detecting ≥30% of PD ≥ 4 mm was 54.5 and for detecting ≥20% of BOP was 35.3.

**Conclusions:**

The data can promote an application of the measurements of lactoferrin and *α*1-antitrypsin in GRF to clinical practice on periodontal disease.

## 1. Introduction

Periodontal disease is prevalent (up to 90%) across countries [1]. This disease, often caused by bacterial invasion, promotes the attachment of connective tissue and the protection of bone around the teeth at the early step of disease, but its subsequent formation of inflammation contributes to the destruction of periodontal tissues [2, 3]. It is thus a chronic inflammatory disorder, which induces not only locally oral but also systemic bodily pathologies [4, 5]. Nowadays, the management of this disease is widely recognized to be crucial.

The diagnosis of periodontal disease has so far relied on human hands to measure the periodontal tissue [6, 7]. Easy and objective measurements using biomarkers are indeed required. Recently, several biomarkers, such as C-reactive protein or bacteria-related DNA/enzyme in saliva and gingival crevicular fluid, have been arisen as the candidates; however, the use of such biomarkers for periodontal disease has not yet to be established [8–10].

The lactoferrin is primarily originated in neutrophils, which response to an acute inflammation [11, 12]. The lactoferrin is enhanced in an anti-inflammatory action through the binding to the lipid A portion of lipopolysaccharide of bacteria [11]. Therefore, lactoferrin is considered an inflammation-related molecule [11, 12]. Also, protease inhibitors are anti-inflammatory reactants, and the *α*1-antitrypsin (a protease inhibitor) derived from serum (i.e., throughout exudate and bleeding) is enhanced by inflammatory cytokines and endotoxins [13, 14]. Therefore, the *α*1-antitrypsin is also considered an inflammation-related molecule [13, 14].

Limited studies (with the different assays) have previously investigated the lactoferrin level or *α*1-antitrypsin level in saliva and in gingival crevicular fluid for periodontal disease and gingival disease [15–19]. An increase of these molecules is suggested to be the potential biomarkers for such diseases [15–19]. Accordingly, the lactoferrin and *α*1-antitrypsin levels using oral materials with an enzyme immunoassay have been measured; the clinical ability of the measurements remains to be determined in daily practice on periodontal disease [20]. The present study investigated the relationship between the periodontal status and these two biomarkers in gingival retention fluid (GRF; a mixture of saliva and gingival crevicular fluid) in comparison to that in saliva (a classical material of this field).

## 2. Patients and Methods

### 2.1. Patients and Sample Measurements

A total of 63 subjects, who visited to clinics for checking the periodontal status, were consecutively enrolled into the current study. Subjects with apparent inflammatory diseases (e.g., respiratory or bowel infection) were excluded. The study was approved by the ethics committee of the Nihon University School of Dentistry (no. EP13D15). Written informed consent was obtained from all participants before their inclusion into the study.

The clinical criteria for periodontal disease (especially periodontitis) were judged from the standard measurements of clinical probing depth. The sampling for lactoferrin and *α*1-antitrypsin in GRF or saliva was performed at the same time. The GRF was collected from maxillary anterior gingiva using a microbrush for 30 seconds before eating any foods [20]. Parafin wax-stimulated whole saliva was collected before clinical examination. Samples were stored in the specific tubes that were applied to measure the lactoferrin and *α*1-antitrypsin levels by an enzyme-link solvent immunoassay using a monoclonal antibody of lactoferrin and *α*1-antitrypsin (which was developed by Ikagaku Co., Ltd. (Kyoto, Japan)) [20]. The coefficient of variation regarding assays was 3.7% in lactoferrin and 2.6% in *α*1-antitrypsin, respectively.

Periodontal disease was evaluated by probing pocket depth (PD) [21]. The PD does not always reflect current periodontal inflammation. The assessment of bleeding on probing (BOP) may be reflective to an active inflammation of periodontal tissue [21]. Specifically, the percentage of sites with a PD ≥ 4 mm was calculated, and clinically moderate periodontitis was defined as ≥30% [22]. Immediately thereafter, the BOP was recorded as present or absent at six sites per tooth. The percentage of sites with BOP was calculated, and clinically active periodontitis was defined at BOP ≥20% [23].

### 2.2. Statistical Analysis

The data are presented as the mean ± standard deviation (for variables with normal distributions), the median and interquartile range (for variables with skewed distributions), or subject number. The difference between the two groups was analyzed by the Student *t*-test. A simple correlation test (Pearson's correlation test) was used to analyze the correlation between variables. A multiple regression analysis was also used to analyze the correlation between variables with adjustment for basic confounders such as age and gender. A receiver operating characteristic (ROC) curve analysis was used to identify cutoff levels of lactoferrin and *α*1-antitrypsin for detecting the outcome. The values of variables with skewed distributions were log transformed in these analyses. A statistical significance (*P* value) was set as <0.05.

## 3. Results

Table 1 shows the clinical data of study subjects. The lactoferrin level was higher in saliva than in GRF. The *α*1-antitrypsin level was also higher in saliva than in GRF. The lactoferrin level in GRF was insignificantly different from the *α*1-antitrypsin level in GRF (*P* > 0.05). The lactoferrin level in saliva was also insignificantly different from the *α*1-antitrypsin level in saliva (*P* > 0.05).

Table 2 shows the simple correlation of lactoferrin in GRF or saliva with other variables. There was a significantly positive correlation of lactoferrin between GRF and saliva (*r* = 0.43, *P* < 0.01), and the correlation remained to show the same trend after adjusting age and gender (*β* = 0.42, *P* < 0.01). There was a significantly positive correlation between the prevalence of PD ≥ 4 mm and lactoferrin in GRF or saliva. These correlations showed the similar trend after adjusting age and gender (GRF: *β* = 0.29, *P* = 0.03, saliva: *β* = 0.42, *P* < 0.01). Also, there was a significantly positive correlation between BOP and lactoferrin in GRF or in saliva. These correlations showed the same trend after adjusting age and gender (GRF: *β* = 0.23, *P* = 0.08, saliva: *β* = 0.41, *P* < 0.01). Finally, there was a significantly positive correlation between lactoferrin and *α*1-antitrypsin, and the correlation was relatively high between lactoferrin and *α*1-antitrypsin in GRF or between lactoferrin and *α*1-antitrypsin in saliva.

Table 3 shows the simple correlation of *α*1-antitrypsin in GRF or saliva with other variables. There was a significantly positive correlation of *α*1-antitrypsin between GRF and saliva (*r* = 0.53, *P* < 0.01), and the correlation remained to show the same trend after adjusting age and gender (*β* = 0.52, *P* < 0.01). There was a significantly positive correlation between the prevalence of PD ≥ 4 mm and *α*1-antitrypsin in GRF or in saliva. These correlations showed the same trend after adjusting age and gender (GRF: *β* = 0.33, *P* = 0.02, saliva: *β* = 0.53, *P* < 0.01). Also, there was a significantly positive correlation between BOP and *α*1-antitrypsin in GRF or in saliva. These correlations showed the same trend after adjusting age and gender (GRF: *β* = 0.39, *P* < 0.01, saliva: *β* = 0.57, *P* < 0.01).

**Figure 1:**
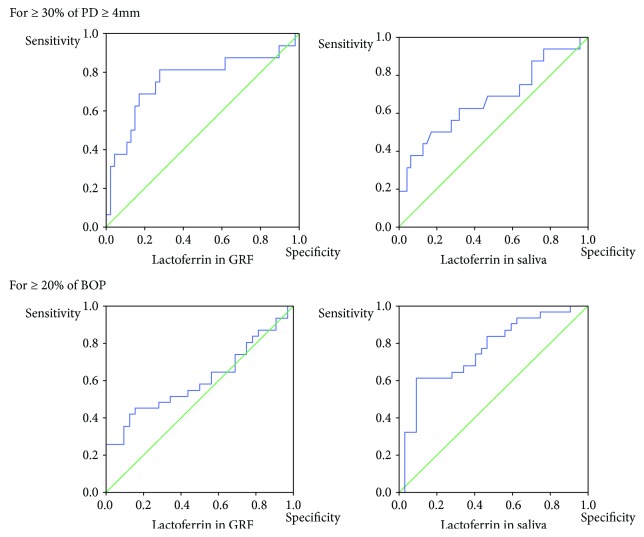
ROC curve analysis of lactoferrin in GRF or in saliva.

Table 4 and Figure 1 show the ROC curve analysis of lactoferrin in GRF or in saliva. The area under the curve (AUC) indicated a significantly moderate accuracy for ≥30% of PD ≥ 4 mm (moderate periodontitis), and the cutoff value (ng/mL) for detecting ≥30% of PD ≥ 4 mm was 68.6 in GRF and 7585.8 in saliva. The AUC of lactoferrin in saliva indicated a significantly moderate accuracy, while the AUC of lactoferrin in GRF indicated a relatively low accuracy for ≥20% of BOP (clinically active periodontitis). The cutoff value (ng/mL) for detecting ≥20% of BOP was 61.2 in GRF and 3715.4 in saliva.

**Figure 2:**
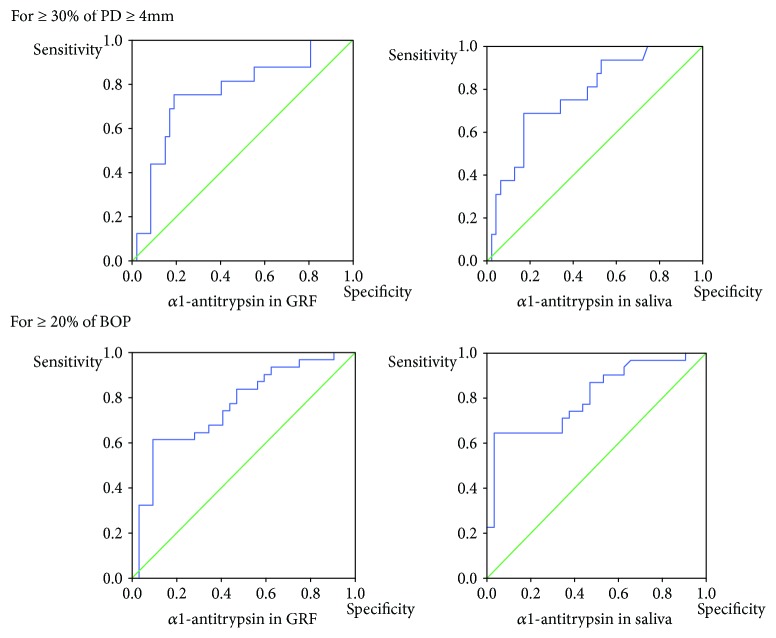
ROC curve analysis of *α*1-antitrypsin in GRF or in saliva.

Table 5 and Figure 2 show the ROC curve analysis of *α*1-antitrypsin in GRF or in saliva. Overall, the AUC of *α*1-antitrypsin appeared to be high relative to that of lactoferrin, while the accuracies of AUC of *α*1-antitrypsin were also at moderate levels for outcomes. The AUC indicated a significantly moderate accuracy for ≥30% of PD ≥ 4 mm, and the cutoff value (ng/mL) for detecting ≥30% of PD ≥ 4 mm was 54.5 in GRF and 8871.6 in saliva. The AUC indicated a significantly moderate accuracy for ≥20% of BOP, and the cutoff value (ng/mL) for detecting ≥20% of BOP was 35.3 in GRF and 4265.8 in saliva.

## 4. Discussion

The present study is the first to investigate clinically the relationships among the periodontal status, lactoferrin, and *α*1-antitrypsin in GRF, in a comparative manner of their relationships in saliva, using an enzyme immunoassay. Saliva is a classical material of this research field, while the use of GRF is reasonable as it is close to periodontal disease. The conventional GCF (gingival crevicular fluid) is collected from the gingival sulcus with one tooth using a paper point. The sampling method using the microbrush used in this study is different from the original GCF. Therefore, we defined newly as GRF (gingival retention fluid). Saliva reflects the entire oral cavity, whereas GCF is considered to reflect the gingival condition of each tooth. However, sampling of GCF is time-consuming and requires certain skills. In contrast, GRF reflects a wider range of gingival conditions, and simple sampling methods can be applied to mass screening. The results of the present study would be valuable to offer the insight in an application of measurements of lactoferrin and *α*1-antitrypsin with the use of GRF to clinical practice on periodontal disease.

The first finding of this study is a moderate correlation between lactoferrin and *α*1-antitrypsin in GRF or in saliva in this population. The two biomarkers are both inflammation-related molecules [11–14], and their increase in periodontal and gingival disease has been previously reported [15–19]. Therefore, the correlation appears to be natural, even though these can have a different pathophysiological origin [11–14]. The overlapping and/or independent application of these biomarkers to clinics is a next issue.

The second finding is a mild-to-moderate correlation in lactoferrin or *α*1-antitrypsin between GRF and saliva, while a higher level of the biomarkers in saliva than in GRF. This appeared to be simply reflective to the difference in the amount of sampled materials.

The third finding (from the results of correlation and ROC curve analyses) is a positively mild-to-moderate accuracy of the lactoferrin level with the severity of periodontal status, as well as the *α*1-antitrypsin level with the severity of periodontal status, in GRF or saliva. These may mean that the measurements of lactoferrin and *α*1-antitrypsin in GRF are available for the diagnosis of periodontal disease. The present study newly provided the cutoff levels on the severity of periodontal status in lactoferrin and *α*1-antitrypsin. Their diagnostic abilities did not necessarily seem to be very high but were moderate, indicating that it could be useful to apply the assays to clinics as a supplemental tool. Under this situation, the lactoferrin in GRF weakly distinguished the severities by BOP. As the BOP is an indicator of active inflammation with exudate and bleeding, the *α*1-antitrypsin (a molecule derived from serum) can distinguish the severities by BOP relative to the lactoferrin, especially in case of the use of GRF. Whether the use of *α*1-antitrypsin is superior to that of lactoferrin in GRF in a specific condition like active inflammation merits a further confirmation. We are now investigating the change in the measurement value due to the improvement of clinical symptoms after treatment.

There was a limitation to the present study. The subject numbers studied were relatively small. The inflammatory molecules in blood and/or the additional inflammatory molecules in GRF were not measured. The microbrush to collect samples was manually operated, and its operation might not completely be standardized.

## 5. Conclusions

The present study demonstrated that the lactoferrin and *α*1-antitrypsin in GRF were positively related to the severity of periodontal status. The measurements of these biomarkers can be applied to clinical practice on periodontal disease, while more multifaced studies are warranted.

## Figures and Tables

**Table 1:** Clinical data of the study subjects.

Variable	Levels
Age, years	48 ± 16
Gender (men/women), number	33/30
Prevalence of PD ≥ 4 mm (%)	10.5 (1.1–30.9)
Subjects with ≥30% of PD ≥ 4 mm, number (%)	16 (25%)
BOP (%)	19.8 (10.5–45.8)
Subjects with ≥20% of BOP, number (%)	31 (49%)
Lactoferrin in GRF (ng/mL)	61.0 (33.8–117.8)^a^^∗∗^
Lactoferrin in saliva (ng/mL)	3611.9 (2789.1–7751.2)^a^^∗∗^
*α*1-antitrypsin in GRF (ng/mL)	54.7 (23.2–212.5)^b^^∗∗^
*α*1-antitrypsin in saliva (ng/mL)	4573.3 (2122.0–10834.1)^b^^∗∗^

PD: probing pocket depth, BOP: bleeding on probing, GRF: gingival retention fluid. The data are presented as the mean ± standard deviation, median (interquartile range), or patient number (%). Significance level (gingival sulcus vs. saliva; ^a^lactoferrin, ^b^*α*1-antitrypsin): ^∗∗^ *P* < 0.01.

**Table 2:** Correlation of lactoferrin in GRF or saliva with variables.

Variable	GRF	Saliva
Age	−0.03 (0.84)	0.11 (0.38)
Male gender	0.20 (0.12)	0.11 (0.38)
Prevalence of PD ≥ 4 mm	0.29 (0.02^∗^)	0.43 (<0.01^∗∗^)
BOP	0.25 (0.047^∗^)	0.42 (<0.01^∗∗^)
Lactoferrin in saliva	0.43 (<0.01^∗∗^)	—
*α*1-antitrypsin in GRF	0.61 (<0.01^∗∗^)	0.39 (<0.01^∗∗^)
*α*1-antitrypsin in saliva	0.44 (<0.01^∗∗^)	0.69 (<0.01^∗∗^)

PD: probing pocket depth, BOP: bleeding on probing, GRF: gingival retention fluid. The data are presented as correlation coefficient *r* (*p*-value) by simple correlation test (Pearson test). Significance level: ^∗^*P* < 0.05, ^∗∗^*P* < 0.01.

**Table 3:** Correlation of *α*1-antitrypsin in GRF or in saliva with variables.

Variable	GRF	Saliva
Age	0.16 (0.22)	0.04 (0.73)
Male gender	0.12 (0.36)	−0.03 (0.80)
Prevalence of PD ≥ 4 mm	0.36 (<0.01^∗∗^)	0.46 (<0.01^∗∗^)
BOP	0.42 (<0.01^∗∗^)	0.50 (<0.01^∗∗^)
*α*1-antitrypsin in saliva	0.53 (<0.01^∗∗^)	—

PD: probing pocket depth, BOP: bleeding on probing, GRF: gingival retention fluid. The data are presented as correlation coefficient *r* (*p*-value) by simple correlation test (Pearson test). Significance level: ^∗^*P* < 0.05, ^∗∗^*P* < 0.01.

**Table 4:** ROC curve analysis of lactoferrin in GRF or saliva.

Outcomes	AUC (95% CI)	*P* value	Cutoff (ng/mL)	Sensitivity	Specificity	PLR	NLR
For ≥30% of PD ≥ 4 mm							
Lactoferrin in GRF	0.76 (0.60–0.92)	<0.01^∗∗^	68.6	0.81	0.72	2.9	0.3
Lactoferrin in saliva	0.67 (0.50–0.84)	0.04^∗^	7585.8	0.50	0.83	2.9	0.3
For ≥20% of BOP							
Lactoferrin in GRF	0.60 (0.46–0.75)	0.16	61.2	0.55	0.56	1.3	0.8
Lactoferrin in saliva	0.70 (0.57–0.83)	<0.01^∗∗^	3715.4	0.61	0.72	3.6	0.5

ROC: receiver operating characteristic, PD: probing pocket depth, BOP: bleeding on probing, GRF: gingival retention fluid, AUC: area under the curve, CI: confidence interval, PLR: positive likelihood ratio, NLR: negative likelihood ratio. Significance level: ^∗^ *P* < 0.05, ^∗∗^ *P* < 0.01.

**Table 5:** ROC curve analysis of *α*1-antitrypsin in GRF or in saliva.

Outcomes	AUC (95% CI)	*P* value	Cutoff (ng/mL)	Sensitivity	Specificity	PLR	NLR
For ≥30% of PD ≥ 4 mm							
*α*1-antitrypsin in GRF	0.76 (0.62–0.90)	<0.01^∗∗^	54.5	0.81	0.60	2.0	0.5
*α*1-antitrypsin in saliva	0.77 (0.65–0.90)	<0.01^∗∗^	8871.6	0.69	0.83	4.0	0.2
For ≥20% of BOP							
*α*1-antitrypsin in GRF	0.76 (0.64–0.88)	<0.01^∗∗^	35.3	0.84	0.53	1.8	0.6
*α*1-antitrypsin in saliva	0.81 (0.70–0.92)	<0.01^∗∗^	4265.8	0.74	0.63	2.0	0.5

ROC: receiver operating characteristic, PD: probing pocket depth, BOP: bleeding on probing, GRF: gingival retention fluid, AUC: area under the curve, CI: confidence interval, PLR: positive likelihood ratio, NLR: negative likelihood ratio. Significance level: ^∗^*P* < 0.05, ^∗∗^*P* < 0.01.

## Data Availability

The authors may make data available on request through the authors themselves. In this case, they should name who should be contacted to request the data and provide appropriate contact details. The provision of data can be needed to be reviewed in the institutional ethics committee.
